# Cryptanalysis of an Image Encryption Algorithm Based on a 2D Hyperchaotic Map

**DOI:** 10.3390/e24111551

**Published:** 2022-10-28

**Authors:** Chengrui Zhang, Junxin Chen, Dongming Chen

**Affiliations:** 1 Software College, Northeastern University, Shenyang 110169, China; 2 School of Software, Dalian University of Technology, Dalian 116621, China

**Keywords:** image encryption, permutation, diffusion, chosen-plaintext attack, chosen-ciphertext attack, 37N99

## Abstract

Recently, an image encryption scheme based on a 2D hyperchaotic map is proposed. It adopts the permutation–diffusion architecture and consists of three steps, which are permutation, forward diffusion, and backward diffusion. In this paper, we break this cipher with both the chosen-plaintext attack (CPA) and the chosen-ciphertext attack (CCA). According to our analysis, we found the two complex diffusion processes could be simplified into two simple diffusions and a modular addition operation. Based on this, the equivalent key can be obtained with CPA and CCA. Detailed theoretical derivations and the results of experiments confirmed the feasibility of our attack methods. When the image size was 256×256, the running time of the attacks was less than 2 hours on a laptop with a 2.59 GHz Intel Core i7 and 16 GB DDR3 memory. Other sizes of images were also tested, and some rules were found. In addition, the probability of other attacks has also been discussed, and some suggestions for improvements are given. The source codes are publicly available and can be found online.

## 1. Introduction

With the dramatic developments of communication technology, we have witnessed in recent years increasing popularity of secure transmission of multimedia data. The image is a common data model, and cryptographic methods are of critical importance for information sharing. Unfortunately, images are different from text in terms of their inherent properties, such as the large data volume and high redundancy [1,2,3]. If we use the traditional textual encryption methods such as 3DES and AES to encrypt images, it tends to lead to low efficiency and cannot fulfill the requirement of real-time transmission. Therefore, many encryption methods specifically for images have been proposed [4,5].

Due to the features of initial value sensitivity, ergodicity, and random similarity [6,7], chaotic systems are able to meet the needs of encryption processes. Many encryption schemes based on chaos have been proposed [8,9,10,11,12]. Fridrich proposed the first permutation–diffusion architecture image encryption scheme in 1998 [1], which became a paradigm adopted by other people. Some encryption schemes combined various mathematical theories of interesting problems [13,14]; for example, Josephus Problem was employed in [13] for designing a secure and efficient image encryption scheme. Some encryption schemes combine DNA coding technologies to acquire the confusion effect similarly to S-box [15,16,17]. Most schemes encrypt the image as a whole, but a few methods divide the image into several small blocks for encryption; e.g., Zhang et al. [18] decomposed the plaintext image into two components, achieving faster encryption speed and higher security. Most of these methods are a combination of permutation and diffusion operations, but there exist a few permutation-only methods, such as [19].

Although many image encryption schemes, to the best of our knowledge, have been proposed in the past few decades, so far no one has matched the security and applicability of AES, which is a widely accepted approach. In fact, many of them fail to meet actual encryption requirements and have been proven to be less secure than was thought when they were proposed [20]. A typical image encryption method [21] based on chaos which consists of two rounds of permutation–diffusion operations was cryptanalyzed by Chen et al. [22]. They found the relationship between the plaintext images and ciphertext images and proposed a chosen-plaintext attack. Some algorithms combined with DNA operations were attacked not long after they were proposed, e.g., [23]. The opponent used the chosen-plaintext attack [24] which regarded the complex DNA operations as the S-box and got the equivalent encryption elements. We can see a similar idea in [25,26]. More similar works can be seen in [27,28,29,30,31].

Permutation and diffusion are two operations that are commonly used in image encryption schemes [32]. The permutation process only scrambles the position of the plaintext pixels, whereas the diffusion changes the value of pixels to obtain an avalanche effect. In 2010, Fridrich’s premutation was cryptanalyzed by Solak et al. [33]. From then on, a series of studies have proven that most of the proposed permutation-only encryption algorithms are insecure against plaintext attacks. Zhang et al. [34] proposed an attack method making the tradeoffs between time and memory. Jolfaei et al. [2] minimized the required conditions by taking advantage of the pigeon nest principle. Accordingly, the diffusion operation was also analyzed in [35,36]. Chen et al. [37] further improved the efficiency of the attack. The general cryptanalysis of permutation–diffusion architecture image algorithm can be found in [38,39]. In addition, there can be different attacks for different encryption schemes. As it turns out, it is not enough to evaluate the security level of an image cipher by the number of pixels change rate (NPCR) and the unified average changing intensity (UACI), etc.

Recently, a novel image encryption algorithm based on 2D hyperchaotic map was proposed by Gao et al. [40]. The authors introduced a new 2D hyperchaotic map which had more complex chaotic behaviors than other chaotic systems. NPCR and UACI were tested to value the security level of the scheme. The encryption scheme consists of three steps, which are permutation, forward diffusion, and backward diffusion. However, according to our analysis, the two complex diffusion processes could be regarded as the combinations of two simple diffusion processes and a modular addition operation. Considering the diffusion processes, only a plaintext–ciphertext pair is sufficient to obtain the equivalent key. Based on this, we found it is vulnerable to both chosen-plaintext attacks and chosen-ciphertext attacks. Theoretical deductions and the results of experiment confirmed the feasibility of our attacks.

Our contributions are summarized as follows.

- An image encryption scheme was broken by both CCA and CPA.
- The equivalent process of original encryption algorithm is given.
- Detailed theoretical derivations and experimental results are given to confirm the feasibility of our approaches.
- The probabilities of other attack methods and some suggestions for improvements are also given.

The remainder of this paper is organized as follows. The second section briefly introduces the original image encryption scheme. Section 3 shows the theoretical analysis and derivation. Section 4 presents the equivalent process of the original image encryption scheme and the details of attacks. Experimental results are provided in Section 5. The possibility of other attacks and some suggestions for improvements are also discussed in Section 5. Finally, the last section concludes this work.

## 2. The Original Image Encryption Scheme

We briefly summarize the encryption scheme, and interested readers can find more details in [40].

### 2.1. Notation

Most of the notation adopted is listed in Table 1. Complementary descriptions are described as follows:

- Generally, *P* and *C* denote the plaintext and ciphertext in an encryption scheme, respectively, and *S* refers to the intermediate result in a process. The plaintext image *P* is assumed to have a size of H×W.
- The modular subtraction of two plaintext images is defined as their differential, denoted as ΔP.
- The notation *T* represents the equivalent key matrix of the diffusion process. The notation SC represents the intermediate result of the target plaintext image and ciphertext image.

### 2.2. The Encryption Procedures

**Figure 1:**
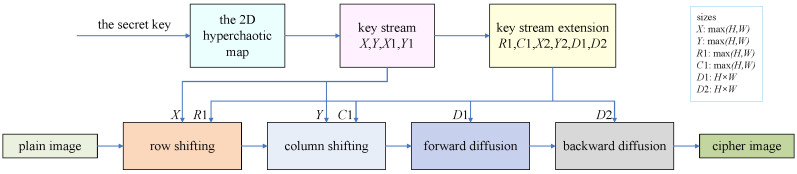
Diagram of the encryption process.

The encryption scheme under study is a permutation–diffusion architecture. It consists of four steps: row shifting, column shifting, forward diffusion and backward diffusion. The row and column shifting can be considered as permutation operations, and the subsequent operations are diffusion. Figure 1 is an overall flowchart of the encryption scheme. The function max(x,y) returns the greater number of *x* and *y*. The secret key generates encryption elements through the chaotic system for the encryption process.

**Figure 2:**
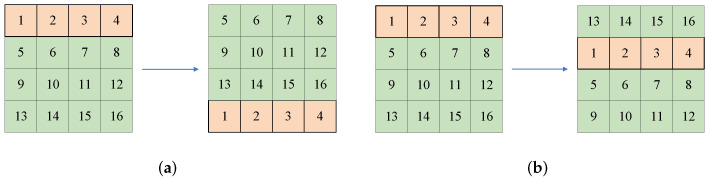
The permutation principle of rows: (**a**) the permutation principle of the 4×4 image when R1(i)=1 and X(i)>0; (**b**) the permutation principle of 4×4 image when R1(i)=1 and X(i)<0.

**Figure 3:**
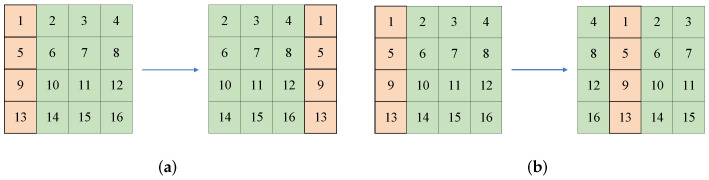
The permutation principle of columns: (**a**) the permutation principle of the 4×4 image when C1(i)=1 and Y(i)>0; (**b**) the permutation principle of 4×4 image when C1(i)=1 and Y(i)<0.

1. Generation of the key stream.
  (a) Iterate the following 2D hyperchaotic system, i.e., Equation (1), (m+N) times to obtain the secret key flow sequence *X* and *Y* of the permutation process, where *N* is the greater number of *H* and *W*. The sequences *X* and *Y* discarded the first *m* values to get the more stable chaotic sequences. The parameters and initial values of the 2D hyperchaotic map, which include h,r,X(0) and Y(0), are the secret keys of the encryption scheme, where h∈[3,7] and r∈[2,6]. In the original encryption paper, the author used h=5,r=5,X(0)=0.5, and *Y*(0) = 0.5. Interested readers can find more information about the 2D hyperchaotic map in [40,41,42].
    (1) $$\left\{\begin{array}{c} X\left(i\right)=sin\left(\frac{h\pi }{sinY(i-1)}\right)\\ Y(i)=rsin(\pi X(i-1)Y(i-1)) \end{array}\right..$$
  (b) Vectors R1 and C1 for permutation are generated by *X* and *Y* through Equation (2). The function floor() rounds the original number to the nearest integer less than or equal to it.
    (2) $$\left\{\begin{array}{c} R1\left(i\right)=\left(\mathrm{floor}(|X\left(i\right)\right|\times 10^{16}))\;\mathrm{mod}\;\frac{H}{2}\\ C1\left(i\right)=\left(\mathrm{floor}(|Y\left(i\right)\right|\times 10^{16}))\;\mathrm{mod}\;\frac{W}{2} \end{array}\right..$$
  (c) Iterate the same chaotic system, i.e., Equation (1), (n+H×W) times to obtain the secret key flow sequence X1 and Y1. The sequences X1 and Y1 discard the first *n* values to get the more stable chaotic sequences. The sequences X2 and Y2 are then obtained by Equation (3). The function round() rounds the original number to the nearest integer.
    (3) $$\left\{\begin{array}{c} X2\left(i\right)=(\mathrm{round}(1000(|X1\left(i\right)\times 10^{16}|-\mathrm{floor}(|X1\left(i\right)|\times 10^{16}))))\;\mathrm{mod}\;256\\ Y2\left(i\right)=(\mathrm{round}(1000(|Y1\left(i\right)\times 10^{16}|-\mathrm{floor}(|Y1\left(i\right)|\times 10^{16}))))\;\mathrm{mod}\;256 \end{array}\right..$$
  (d) Finally, we can get the matrixes D1 and D2 for forward and backward diffusion from X2 and Y2 by Equation (4). The function reshape() is used to change the size of a matrix. If the input is a matrix of size M×N and the output is a matrix of size H×W, then it must satisfy M×N=H×W. The elements of the matrix are read by column and stored by column. The function reshape (X,M,N) returns the *M*-by-*N* matrix whose elements are taken column-wise from *X*.
    (4) $$\left\{\begin{array}{c} D1=\mathrm{reshape}(X2,(H,W))\\ D2=\mathrm{reshape}(Y2,(H,W)) \end{array}\right..$$
2. Row shifting
  The image *P* is row-permuted with chaotic sequences *X* and R1. The permutation principles are shown in Figure 2. For the input image with size 4×4, R1(i)=1, when X(i)>0, the principle is shown in Figure 2a; for X(i)<0, the process is shown in Figure 2b.
3. Column shifting Similarly, the column-permuted result is obtained by chaotic sequences *Y* and C1. The permutation principles are shown in Figure 3. For the input image with size 4×4, C1(i)=1, when Y(i)>0, the principle is shown in Figure 3a; when Y(i)<0, the process is shown in Figure 3b.
4. Forward diffusion
  With the help of D1 generated before, the forward diffusion is implemented according to Equation (5), in which S2 is the forward diffusion result and S1 represents the input image after row and column shifting, i=2,3,4,⋯,H; j=2,3,4,⋯,W.
  (5) $$\left\{\begin{array}{c} S2(1,1)=(S1(1,1)+D1(1,1))\;\mathrm{mod}\;256\\ S2(1,j)=(S1(1,j)+D1(1,j)+S2(1,j-1))\;\mathrm{mod}\;256\\ S2(i,1)=(S1(i,1)+D1(i,1)+S2(i-1,1))\;\mathrm{mod}\;256\\ S2(i,j)=(S1(i,j)+D1(i,j)+S2(i,j-1)+S2(i-1,j))\;\mathrm{mod}\;256 \end{array}\right..$$
5. Backward diffusion Similarly, the backward diffusion process is performed with the matrix D2, which is generated before. The diffusion operation is described in Equation (6), where *C* is the final encryption result; i=H−1,H−2,⋯,1; j=W−1,W−2,⋯,1.
  (6) $$\left\{\begin{array}{c} C(H,W)=(S2(H,W)+D2(H,W))\;\mathrm{mod}\;256\\ C(H,j)=(S2(H,j)+D2(H,j)+C(H,j+1))\;\mathrm{mod}\;256\\ C(i,W)=(S2(i,W)+D2(i,W)+C(i+1,W))\;\mathrm{mod}\;256\\ C(i,j)=(S2(i,j)+D2(i,j)+C(i,j+1)+C(i+1,j))\;\mathrm{mod}\;256 \end{array}\right..$$

## 3. Security Analysis

The original encryption scheme has two main steps, which are permutation and diffusion. The secret key generates the key stream at each step through a novel chaotic system. For the permutation phase, we use a permutation matrix as the equivalent key. For the diffusion phase, the equivalent keys are two matrices. According to our analysis, it is enough to use only one matrix as the equivalent key in the diffusion phase.

Before describing the attack method, we give some necessary conclusions and prove them.

**Theorem** **1.** *If we only consider the diffusion phase of the encryption scheme, each pixel value of the ciphertext is the sum of the pixel values of the plaintext and the corresponding location value of the equivalent key matrix, which is given by*

(7) $$C\left(i,j\right)=(f_{P}\left(i,j\right)+T\left(i,j\right))\;\mathrm{mod}\;256,$$

*where $f_{p}\left(i,j\right)$ represents a variable that is related to the values of plaintext and its location and T(i,j) represents the corresponding value of the equivalent key matrix T which is dependent on the secret key.*

**Proof of Theorem** **1.** We prove the forward diffusion process, which has four diffusion principles. The backward diffusion is symmetric with the forward diffusion and has the same theorem.

**Figure 4:**
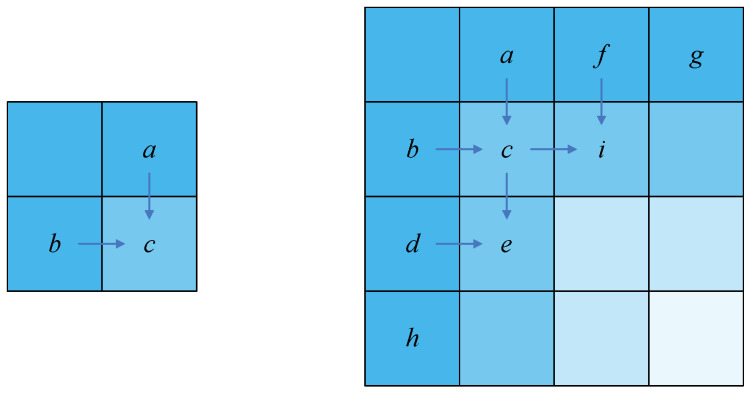
Schematic diagram of induction.

1. For the first formula of the forward diffusion phase, i.e., the first subformula of Equation (5), we are able to obtain
  (8) C(1,1)=(P(1,1)+D1(1,1))mod256.
  Obviously, Equation (8) satisfies the form of Equation (7). So the first value of the pixel satisfies Equation (7).
2. For the second formula of the forward diffusion phase, it is derived by:
  $$\begin{array}{cc} C(1,j) & =(P(1,j)+D1(1,j)+C(1,j-1))\;\mathrm{mod}\;256\\ C(1,j-1) & =(P(1,j-1)+D1(1,j-1)+C(1,j-2))\;\mathrm{mod}\;256\\ \cdots \\ C(1,2) & =(P(1,2)+D1(1,2)+C(1,1))\;\mathrm{mod}\;256. \end{array}$$
  If we add both sides of this equation, we get
  (9) $$C\left(1,j\right)=(\sum_{j=2}^{j}\left(P\left(1,j\right)+D1\left(1,j\right)\right)+C\left(1,1\right))\;\mathrm{mod}\;256.$$
  Substitute Equation (8) into Equation (9). We obtain Equation (10).
  (10) $$\begin{array}{cc} C(1,j) & =(\sum_{j=2}^{j}P\left(1,j\right)+P\left(1,1\right)+\sum_{j=2}^{j}D1\left(1,j\right)+D1\left(1,1\right))\;\mathrm{mod}\;256\\ \; & =(\sum_{j=1}^{j}P\left(1,j\right)+\sum_{j=1}^{j}D1\left(1,j\right))\;\mathrm{mod}\;256. \end{array}$$
  It was found that Equation (10) also satisfies the form of Equation (7). In other words, the values of the first row satisfy Equation (7).
3. The derivation of the third part is very similar to that of the second stage. For the third formula of forward diffusion, it is deduced from:
  $$\begin{array}{cc} C(i,1) & =(P(i,1)+D1(i,1)+C(i-1,1))\;\mathrm{mod}\;256\\ C(i-1,1) & =(P(i-1,1)+D1(i-1,1)+C(i-2,1))\;\mathrm{mod}\;256\\ \cdots \\ C(2,1) & =(P(2,1)+D1(2,1)+C(1,1))\;\mathrm{mod}\;256. \end{array}$$
  Similarly, we can conclude
  (11) $$\begin{array}{cc} C(i,1) & =(\sum_{i=2}^{i}\left[P\left(i,1\right)+D1\left(i,1\right)\right]+C\left(1,1\right))\;\mathrm{mod}\;256\\ \; & =(\sum_{i=1}^{i}P\left(i,1\right)+\sum_{i=1}^{i}D1\left(i,1\right))\;\mathrm{mod}\;256. \end{array}$$
  We were able to find that Equation (11) also satisfies the form of Equation (7). In other words, the values of the first column satisfy Equation (7).
4. In this section, we use a method which is similar to mathematical induction. As shown in Figure 4, if we assume that both pixel values, *a* and *b*, satisfy Equation (7), which is able to deduce that the value *c* also satisfies Equation (7), then if both the pixels of the first row and first column satisfy Equation (7), we can deduce that all the pixels satisfy Equation (7). It seems that this theorem will be transferred from the left and upper pixels to the middle pixels.
  If we suppose Equations (12) and (13) are correct and substitute Equations (12) and (13) into the last formula of forward diffusion, we can obtain Equation (14).
  (12) $$C\left(i,j-1\right)=(f_{p1}\left(i,j\right)+T1\left(i,j\right))\;\mathrm{mod}\;256,$$
  (13) $$C\left(i-1,j\right)=(f_{p2}\left(i,j\right)+T2\left(i,j\right))\;\mathrm{mod}\;256.$$
  (14) $$\begin{array}{cc} C(i,j) & =(P(i,j)+D1(i,j)+C(i,j-1)+C(i-1,j))\;\mathrm{mod}\;256\\ \; & =(P\left(i,j\right)+f_{p1}\left(i,j\right)+f_{p2}\left(i,j\right)+D1\left(i,j\right)+T1\left(i,j\right)+T2\left(i,j\right)\;\mathrm{mod}\;256\\ \; & =(f_{p3}\left(i,j\right)+T3\left(i,j\right))\;\mathrm{mod}\;256. \end{array}$$
  From Equation (14), we can deduce that if we suppose the pixel C(i,j−1) and pixel C(i−1,j) satisfy Equation (7), then pixel C(i,j) satisfies Equation (7). These pixels correspond to the pixels *a*, *b*, and *c* of Figure 4.
  With i=2 and j=2, the pixels C(1,2) and C(2,1) satisfyEquation (7). Both the pixels of the first row and the first column satisfy Equation (7), which can be derived from the previous proof. Therefore, all the pixels of the image satisfy Equation (7).

   □

**Theorem** **2.** *If we only consider the diffusion phase, the differential of ciphertexts is related to the differential of plaintexts. More specifically, the module subtraction of the pixel value of the ciphertext images is the module subtraction of the plaintext images after the diffusion process without encryption parameters.*

**Proof of Theorem** **2.** From the conclusion in Theorem 1, we can obtain

(15) $$\begin{array}{cc} (C_{1}\left(i,j\right)-C_{2}\left(i,j\right))\;\mathrm{mod}\;256 & =(\left(f_{P1}\left(i,j\right)+T\left(i,j\right)\right)-\left(f_{P2}\left(i,j\right)+T\left(i,j\right)\right)\;\mathrm{mod}\;256\\ \; & =(f_{P1}\left(i,j\right)-f_{P2}\left(i,j\right))\;\mathrm{mod}\;256.\\ \; & =f_{\Delta P}\left(i,j\right). \end{array}$$

   □

## 4. The Proposed Attack

### 4.1. The Equivalent Processes

According to our analysis, the original encryption algorithm is equivalent to the combination of some simple processes, in which the encryption elements consist of a permutation matrix and an equivalent diffusion matrix.

#### 4.1.1. The Equivalent Encryption Process

The equivalent encryption process comprises four phases, i.e., permutation, simple forward diffusion, simple backward diffusion, and modular addition with the equivalent key matrix.

**Figure 5:**
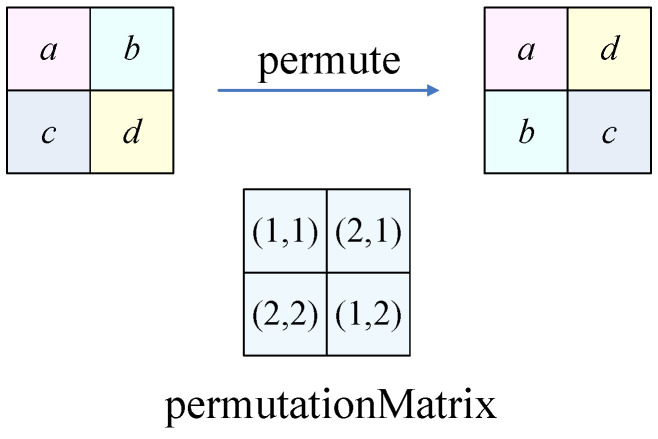
The equivalent permutation.

1. Permutation
  The plaintext image permutes its pixels with the permutation matrix through Equation (16). An illustration is shown in Figure 5.
  (16) S=permute(P,permutationMatrix).
2. Simple forward diffusion
  The simple forward diffusion is implemented according to Equation (17), where S1 is the simple forward diffusion result, *S* represents the input image after permutation, and i=2,3,4,⋯,H; j=2,3,4,⋯,W.
  (17) $$\left\{\begin{array}{c} S1(1,1)=S(1,1)\\ S1(1,j)=(S(1,j)+S1(1,j-1))\;\mathrm{mod}\;256\\ S1(i,1)=(S(i,1)+S1(i-1,1))\;\mathrm{mod}\;256\\ S1(i,j)=(S(i,j)+S1(i,j-1)+S1(i-1,j))\;\mathrm{mod}\;256 \end{array}\right..$$
3. Simple backward diffusion
  Similarly, the simple backward diffusion operation is described in Equation (18), where S2 is the diffusion result; i=H−1,H−2,⋯,1; j=W−1,W−2,⋯,1.
  (18) $$\left\{\begin{array}{c} S2(H,W)=S1(H,W)\\ S2(H,j)=(S1(H,j)+S2(H,j+1))\;\mathrm{mod}\;256\\ S2(i,W)=(S1(i,W)+S2(i+1,W))\;\mathrm{mod}\;256\\ S2(i,j)=(S1(i,j)+S2(i,j+1)+S2(i+1,j))\;\mathrm{mod}\;256 \end{array}\right..$$
4. Modular addition with the equivalent key
  The final ciphertext image *C* will be generated using the diffusion result S2 and the equivalent key matrix *T* through Equation (19).
  (19) C(i,j)=(S2(i,j)+T(i,j))mod256.

#### 4.1.2. The Decryption Process

Corresponding to the equivalent encryption process, the decryption process consists of four steps. They are modular subtraction, inverse backward diffusion, inverse forward diffusion, and inverse permutation.

**Figure 6:**
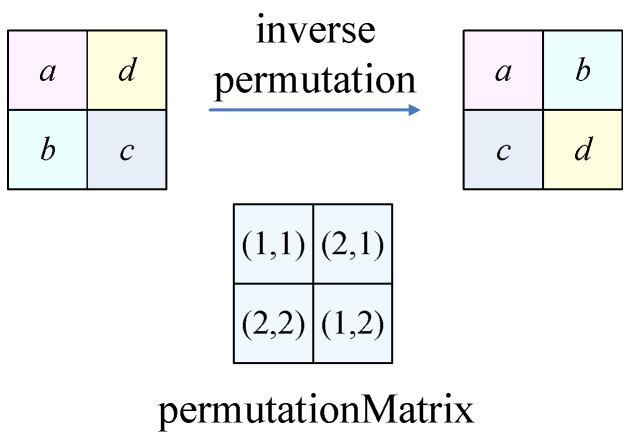
The inverse permutation.

1. Modular subtraction with an equivalent key matrix
  The immediate result *S*2 will be obtained from Equation (20), where *C* represents the final ciphertext image and *T* represents the equivalent key matrix.
  (20) S2(i,j)=(C(i,j)−T(i,j))mod256.
2. Inverse backward diffusion
  The immediate result *S*1 will be obtained from Equation (21), where i=H−1,H−2,⋯,1; j=W−1,W−2,⋯,1.
  (21) $$\left\{\begin{array}{c} S1(H,W)=S2(H,W)\\ S1(H,j)=(S2(H,j)-S2(H,j+1))\;\mathrm{mod}\;256\\ S1(i,W)=(S2(i,W)-S2(i+1,W))\;\mathrm{mod}\;256\\ S1(i,j)=(S2(i,j)-S2(i,j+1)-S2(i+1,j))\;\mathrm{mod}\;256 \end{array}\right..$$
3. Inverse forward diffusion
  The immediate result *S* will be obtained from Equation (22), where i=2,3,4,⋯,H; j=2,3,4,⋯,W.
  (22) $$\left\{\begin{array}{c} S(1,1)=S1(1,1)\\ S(1,j)=(S1(1,j)-S1(1,j-1))\;\mathrm{mod}\;256\\ S(i,1)=(S1(i,1)-S1(i-1,1))\;\mathrm{mod}\;256\\ S(i,j)=(S1(i,j)-S1(i,j-1)-S1(i-1,j))\;\mathrm{mod}\;256 \end{array}\right..$$
4. Inverse permutation
  The plaintext image *P* will be obtained using the intermediate result *S* and permutation matrix from Equation (23). An illustration is shown in Figure 6.
  (23) P=inversePermute(S,permutationMatrix).

### 4.2. The Proposed Chosen-Plaintext Attack

In a chosen-plaintext attack scenario, the attackers can freely select a set of plaintexts and get the corresponding ciphertexts. Then, the information of secret key or encryption elements will be derived by these known plaintext–ciphertext pairs. The attackers use them to recover the target plaintext.

When we take an all-zero image as input, the permutation result is also an all-zero image. Then, we use the images before and after diffusion to obtain the equivalent key at this stage. When encryption elements in the diffusion stage are acquired, the encryption algorithm is simplified into a permutation process. The special plaintext–ciphertext pairs are then constructed to obtain the equivalent permutation matrix. Finally, we recover the plaintext using the previously obtained equivalent key.

Algorithm 1 reveals the process of the proposed chosen-plaintext attack. The details of Algorithm 1 are described below.
**Algorithm 1** The proposed chosen-plaintext attack**Output:** The plaintext *P* of *C***Input:** The ciphertext *C* of size *H* × *D*1:// step1: construct an all-zero matrix and obtain the equivalent key *T* of diffusion stage2:P0 = zeros(H,W);3:C0 = encrypt(P0);4:T=C0;5:// step2: initialize $\left\lceil \log_{L}\left(HW\right)\right\rceil$ matrices and get the corresponding ciphertexts6:**for** i=1 to $\left\lceil \log_{L}\left(HW\right)\right\rceil$ **do**7:   Pi = JolfaeiAlgorithmGeneration(*i*);8:   Ci = encrypt(Pi);9:**end for**10:// step3: obtain $\left\lceil \log_{L}\left(HW\right)\right\rceil$ middle results with the diffusion matrix *T* and the ciphertexts which are obtained in the step211:**for** i=1 to $\left\lceil \log_{L}\left(HW\right)\right\rceil$ **do**12:   Si= inverseDiffusionAttack(Ci−T);13:**end for**14:// step4: obtain the equivalent permutation matrix by the input plaintexts and the middle results15:**for** i=1 to $\left\lceil \log_{L}\left(HW\right)\right\rceil$ **do**16:   JolfaeiAlgorithmAdd(Pi,Si);17:**end for**18:permutationMatrix = JolfaeiAlgorithm();19:// step5: recover the the plaintext of the target ciphertext with the equivalent key stream elements which are obtained in previous steps20:SC = inverseDiffusionAttack(C−T);21:*P* = inversePermute(SC,permutationMatrix);

1. Construct an all-zero matrix and obtain the equivalent key *T*.
  - The permutation process has no effect on an all-zero image, so we can get one pair of input and output of the diffusion phase.
  - According to Theorem 1, i.e., $C\left(i,j\right)=f_{P}\left(i,j\right)+T\left(i,j\right)$, when P0=zeros(H,W), then C0=T.
2. Initialize $\left\lceil \log_{L}\left(HW\right)\right\rceil$ matrices and get the corresponding ciphertexts
  - The function JolfaeiAlgorithmGeneration() is used to generate specially designed plaintexts of JolfaeiAlgorithm(). The function JolfaeiAlgorithmAdd() takes the specially designed plaintext–ciphertext pairs as input to the JolfaeiAlgorithm(). Readers can find more information in Appendix A.
3. Obtain $\left\lceil \log_{L}\left(HW\right)\right\rceil$ middle results with the diffusion matrix *T*.
  - The function inverseDiffusionAttack() refers to the processes of Equations (21) and (22).
4. Obtain the equivalent permutation matrix
  - The function JolfaeiAlgorithm(), i.e., Jolfaei’s algorithm [2], is a chosen-plaintext attack method for permutation-only image encryption algorithms. Interested readers can find more details in Appendix A.
5. Recover the plaintext
  - The function inversePermute() refers to the process of Equation (23).

### 4.3. The Proposed Chosen-Ciphertext Attack

In cryptanalysis, in contrast to chosen-plaintext attack, chosen-ciphertext attackers are able to obtain the plaintexts of the ciphertexts which they specially designed. After that, attackers acquire secret encryption elements or equivalent key streams. The plaintext that attackers wish to recover is retrieved after easily obtaining the necessary information.

For the current encryption algorithm under study, we found that making a differential of the ciphertexts eliminates the equivalent key stream of the diffusion phase. With this property, we can simplify the encryption algorithm to a permutation-only process. We can use the existing algorithm to obtain the equivalent permutation matrix. After obtaining the middle results, we can also obtain the equivalent key of diffusion. When all the necessary information has been collected, we then recover the plaintext as we did in chosen-plaintext method.

Algorithm 2 discloses the process of the proposed chosen-ciphertext attack. The details of Algorithm 2 are described below.
**Algorithm 2** The proposed chosen-ciphertext attack**Input:** The ciphertext *C* of size H×W**Output:** The plaintext *P* of *C*1:// step1: construct $\lceil \log_{L}\left(HW\right)\rceil +1$ ciphertext-plaintext pairs and compute the differentials respectively2:C0 = rand(H,W);3:P0 = decrypt(C0);4:**for** i=1 to $\left\lceil \log_{L}\left(HW\right)\right\rceil$ **do**5:   // construct $\left\lceil \log_{L}\left(HW\right)\right\rceil$ ciphertexts and obtain the corresponding plaintexts6:   ΔSi = JolfaeiAlgorithmGeneration(*i*);7:   ΔCi = diffusionAttack(ΔSi);8:   Ci=C0+ΔCi;9:   Pi = decrypt(Ci);10:   // compute $\left\lceil \log_{L}\left(HW\right)\right\rceil$ differentials of plaintexts11:   ΔPi=Pi−P0;12:**end for**13:// step2: get the equivalent permutation matrix by the differential of plaintexts and intermediate differential results14:**for** i=1 to $\left\lceil \log_{L}\left(HW\right)\right\rceil$ **do**15:   JolfaeiAlgorithmAdd(ΔPi,ΔSi);16:**end for**17:permutationMatrix = JolfaeiAlgorithm();18:// step3: obtain an intermediate result with the permutation matrix19:S0 = permute(P0,permutationMatrix);20:// step4: obtain the equivalent key stream matrix of the diffusion process from the intermediate results and the corresponding ciphertext.21:T=C− diffusionAttack(S0);22:// step5: recover the plaintext of the target ciphertext with the equivalent key stream elements23:SC = inverseDiffusionAttack(C−T);24:*P* = inversePermute(SC,permutationMatrix);

1. Construct $\lceil \log_{L}\left(HW\right)\rceil +1$ ciphertext–plaintext pairs and compute the differentials.
  - The functions diffusionAttack() are the processes of Equations (17) and (18).
2. Get the equivalent permutation matrix
  - The differential of plaintexts and the differential of ciphertexts follow the same permutation principle. Thus, we can use these differentials to obtain the permutation matrix by Jolfaei’s algorithm.
3. Obtain an intermediate result with the permutation matrix.
  - The function permute() is the process of Equation (16).
4. Obtain the equivalent key stream matrix of the diffusion process
  - According to Theorem 1, i.e., $C\left(i,j\right)=f_{P}\left(i,j\right)+T\left(i,j\right)$, when P=S0 and C=∑S0+T, then T=C−∑S0.
5. Recover the plaintext

## 5. Experiments and Discussions

### 5.1. Experimental Results

This section presents the experiment results, which well validate the feasibility of our attack (The test images. Available online: https://sipi.usc.edu/database/database.php?volume=misc (accessed on 20 October 2022)). The proposed attacks and the studied encrytption schemes were implemented by simulations using matlab 2021a on a laptop with specifications of 2.59 GHz Intel Core i7 and 16 GB DDR3 memory, and the source code is openly accessible (The source code. Available online: https://github.com/F-cook/attack_test.git (accessed on 20 October 2022)).

**Figure 7:**
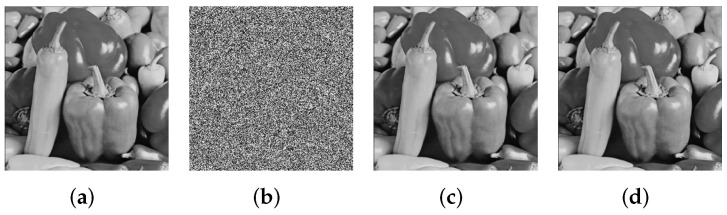
Numerical simulation results: (**a**) the original pepper image of size 256×256 and pixel gray values vary from 0 to 255; (**b**) the encrypted ciphertext image; (**c**) the recovered image using the chosen-plaintext attack; (**d**) the recovered image using the chosen-ciphertext attack.

The experimental results show the plaintext image was recovered successfully. The recovered image is exactly same as the plaintext image. Both CCA and CPA recovered the image without any error. When the image size was 256×256, the running time of CPA was 6776.714213s, and that of CCA was 6754.676951s. The original image is shown in Figure 7a, and the encrypted is shown in Figure 7b. Figure 7c shows the image recovered by CPA, and Figure 7d shows the image recovered by CCA.

**Figure 8:**
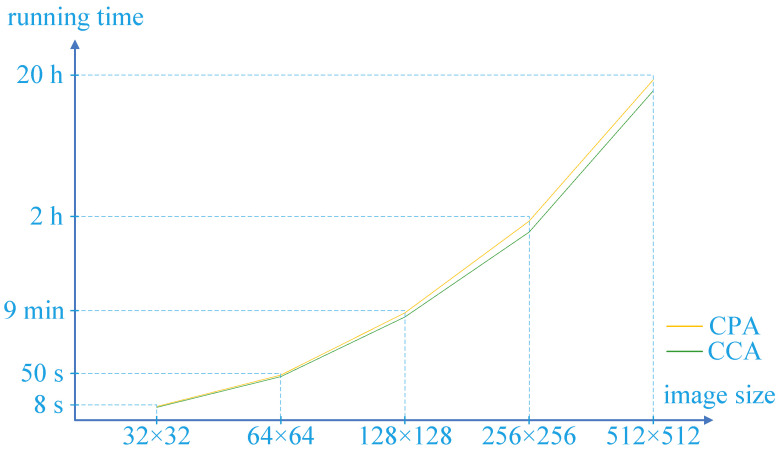
The running times of the CPA and CCA.

In addition, other sizes of images were also tested in our experiments. The running times of our attacks on different sizes of images are shown in Figure 8. All the images which were tested in our experiments are gray images. The original encryption scheme was designed for gray images and regards a color image as three gray images. Therefore, our methods are also applicable to color images, and the attack time on a color image is three times as long as that on a gray image of the same size.

It is worth mentioning that all the experimental results were obtained in our computing environment, which was a laptop with computing specifications of 2.59 GHz Intel Core i7 and 16 GB DDR3 memory. The running time of attack methods varied greatly in different environments.

It is found that as the image size becomes greater, the running time of the CPA and CCA increases rapidly. The CPA is a little faster than CCA. This is because the CCA needs to build more complex inputs to obtain the equivalent secret key.

### 5.2. The Probabilities of Other Attacks

As mentioned above, chosen-plaintext and chosen-ciphertext attacks are effective against the original encryption algorithm, and both attack methods exploit the weaknesses of the scheme. Below we briefly discuss the feasibility of known-plaintext and ciphertext-only attacks.

In the scenario of a known-plaintext attack, the attacker has a set of ciphertexts to which they know the corresponding plaintexts. The attacker cannot control the plaintext–ciphertexts pairs. In this case, random plain-ciphertext pairs often bring redundant information that is helpful in attacking the secret key system. If only the scrambling process is considered, in order to obtain a complete equivalent key stream, an extremely large number of random plaintext and ciphertext pairs are required, which makes it impossible to implement an attack in reality.

If an encryption system is resistant to known-plaintext attacks, then it must be resistant to ciphertext-only attacks. In the scenario of a ciphertext-only attack, the cryptanalyst has access only to a collection of ciphertexts. Attackers can generally guess the ciphertext corresponding to some plaintexts based on some plaintext format information, etc., and infer the secret key information based on the correspondence between very few plaintext ciphertexts. However, for the encryption system analyzed in this article, it is difficult to obtain a small amount of guessed information, and it is impossible to crack the entire encryption system.

### 5.3. Some Suggestions for Improvements

The original encryption scheme is vulnerable to both chosen-plaintext and chosen-ciphertext attacks. The complex encryption can be equivalent to simple processes, and the equivalent key can be obtained by CCA or CPA. In order to improve the security of the original encryption algorithm, some potentially useful suggestions are given below.

- It is suggested that the secret key includes plaintext-related information. The plaintext-dependent ciphers are highly resistant to plaintext attacks. Hash value and the hamming distance are common technologies which have high security.
- Multiple rounds of encryption are recommended. Multiple rounds of encryption help ciphers to achieve a high level of security. Different keys can be used in different rounds, but there should not be too many rounds so as not to affect the encryption efficiency.
- Some random information can be added to the encryption process. Random encryption elements cannot be obtained by various attacks and are very helpful to improve the security level of the encryption scheme.

### 5.4. Limitations and Future Works

Our proposed attack methods, which include CPA and CCA, are special for the original encryption scheme. In other words, they are not universal and cannot be applied to other different image ciphers. However, the idea of obtaining the equivalent key and eliminating the diffusion process may be applied to future cryptanalysis works. In addition, the original encryption scheme may have other flaws which can be used to break it. The permutation process is line by line, which will lead to unrandom results. There may be a more simple way to break the scheme. We only demonstrated the feasibility of our approaches in this paper.

We will explore the more general method to break a family of image encryption schemes. These schemes may have similar encryption processes or common weaknesses. We will use the weaknesses to obtain the equivalent key to break the scheme. More effective attack methods will be proposed. The corresponding suggestions will be also given to resist similar attacks. We believe that better image encryption schemes will be designed in the future.

## 6. Conclusions

In this paper, a permutation–diffusion image encryption based on a 2D hyperchaotic cap was cryptanalyzed, and two attacks, CPA and CCA, were proposed. The equivalent key of the original encryption was obtained, and the transformed process was also described in detail. The theory of the attack method was given by the detailed formula derivation. Different sizes of images were tested in the experiments to demonstrate the feasibility of the two attacks. The other attacks were discussed, and some suggestions for improvements were given. We believe that the ideas of this paper will provide an example of similar cryptanalysis works and give suggestions for the design of image schemes in the future.

## Figures and Tables

**Table 1:** Summary of the notation.

Notation	Description
*A*	generally denotes an matrix, some also represent a value
*A*(*i,j*)**	an element in row *i*, column *j* of a matrix

**Figure A1:**
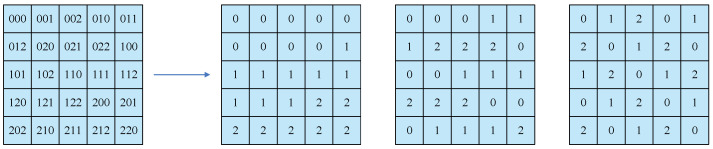
The chosen plaintext images.

**Figure A2:**
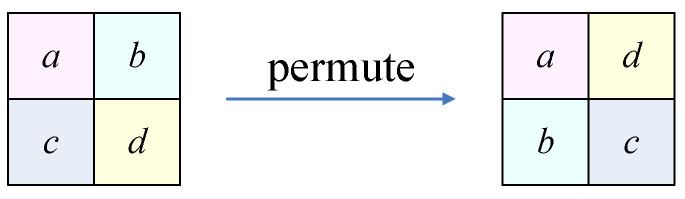
The supposed premutation principle.

**Figure A3:**
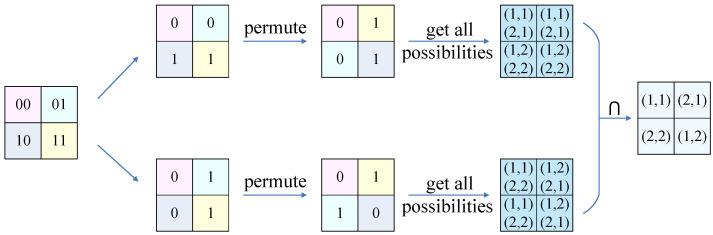
The complete attack process.

## Data Availability

The image data are available in: https://sipi.usc.edu/database/database.php?volume=misc (accessed on 20 October 2022). The source codes are open and can be found in: https://github.com/F-cook/attack_test.git (accessed on 20 October 2022).

## Appendix A. The introduction of Jolfaei’s Algorithm

Jolfaei’s algorithm [2] is a chosen-plaintext attack for permutation-only ciphers. When the size of plaintext image is H×W, the required plaintext–ciphertext pairs are $\left\lceil \log_{L}\left(HW\right)\right\rceil$.

Permutation processes change the positions of pixels of the input image. An image which is of size H×W with *L* different color intensities has H×W locations and at most *L* different pixel values. Thus, one plaintext–ciphertext image pair determines at most *L* positions of an image. The number *n* of plaintext–ciphertext image pairs required to break the permutation-only image cipher must satisfy Equation (A1).

(A1) $$L^{n}\geq HW.$$

Furthermore, we get Equation (A2).

(A2) $$n\geq \log_{L}\left(HW\right).$$

Based on Equation (A2), we found that the number *n* is at least log${}_{L}\left(HW\right)$. To minimize the amount of chosen plain images required, we designed the plaintext with specific rules.

To illustrate the special plaintext design rule, consider a 5×5 image. If the L=3, then the permutation can be determined by $\lceil \log_{3}\left(5\times 5\right)\rceil =3$ plaintext–ciphertext image pairs. The three special plaintext images are shown in Figure A1. The chosen-plaintext images are obtained by splitting the first image into three. The complete permutation rules are obtained by these chosen-plaintext images and their ciphertext images.

Lastly, we utilize an example to illustrate the complete attack procedure. We propose a permutation principle, which is shown in Figure A2, and H=W=L=2. The complete attack process for obtaining the permutation matrix is shown in Figure A3. Firstly, we construct the chosen plaintexts from the plaintext image. Then we obtain the ciphertexts corresponding to the chosen plaintexts. After that, we find all possible cases based on the obtained plaintext–ciphertext pairs. Finally, we obtain the correct scrambling rule by taking the intersection of all possible outcomes.
